# Time course of tolerance to the performance benefits of caffeine

**DOI:** 10.1371/journal.pone.0210275

**Published:** 2019-01-23

**Authors:** Beatriz Lara, Carlos Ruiz-Moreno, Juan José Salinero, Juan Del Coso

**Affiliations:** Camilo José Cela University, Exercise Physiology Laboratory, Madrid, Spain; Norwegian University of Science and Technology, NORWAY

## Abstract

The ergogenic effect of acute caffeine ingestion has been widely investigated; however, scientific information regarding tolerance to the performance benefits of caffeine, when ingested on a day-to-day basis, is scarce. The aim of this investigation was to determine the time course of tolerance to the ergogenic effects of a moderate dose of caffeine. Eleven healthy active participants took part in a cross-over, double-blind, placebo-controlled experiment. In one treatment, they ingested 3 mg/kg/day of caffeine for 20 consecutive days while in another they ingested a placebo for 20 days. Each substance was administered daily in an opaque unidentifiable capsule, and the experimental trials started 45 min after capsule ingestion. Two days before, and three times per week during each 20-day treatment, aerobic peak power was measured with an incremental test to volitional fatigue (25 W/min) and aerobic peak power was measured with an adapted version of the Wingate test (15 s). In comparison to the placebo, the ingestion of caffeine increased peak cycling power in the incremental exercise test by ~4.0 ±1.3% for the first 15 days (*P*<0.05) but then this ergogenic effect lessened. Caffeine also increased peak cycling power during the Wingate test on days 1, 4, 15, and 18 of ingestion by ~4.9 ±0.9% (*P*<0.05). In both tests, the magnitude of the ergogenic effect of caffeine vs. placebo was higher on the first day of ingestion and then progressively decreased. These results show a continued ergogenic effect with the daily ingestion of caffeine for 15–18 days; however, the changes in the magnitude of this effect suggest progressive tolerance.

## Introduction

The ergogenic effects of acute ingestion of caffeine (3–9 mg/kg) have been well contrasted in a variety of exercise situations in original investigations and subsequent meta-analyses [1–4], although other factors such as sex, timing and form of caffeine administration need further research [5]. There is also a growing consensus about the main mechanism behind caffeine ergogenicity during locomotor activities, with much evidence in animal [6, 7] and human models [8] supporting the ability of caffeine to act as an adenosine A_1_ and A_2A_ receptor antagonist. Although other mechanisms, such as fatty acid mobilization and oxidation, potassium ion attenuation in the interstitium and calcium iron release from the sarcoplasmic reticulum have also been proposed [2, 9], the inhibition of the negative effects that adenosine induces on neurotransmission, arousal and pain perception has been proposed as the main mechanism behind the effectiveness of caffeine to increase performance in endurance- and power-based exercises [10, 11]. However, there is controversy about the presence of progressive tolerance to its ergogenic effects when ingested on a day-to-day basis.

It is a common phenomenon that tissues respond to hormonal overstimulation with a decrease in the number of pertinent receptors and to hormonal understimulation with an increase in the number of pertinent receptors [12]. According to this notion, Fredholm [13] found an increase in the number of binding sites for adenosine in the brain cortex of rats treated for two weeks with 10 mg/kg/day of caffeine. This mechanism suggests that the daily intake of caffeine would result in more newly created adenosine receptors, reducing in part the blocking-action of caffeine and thus, its ergogenicity during exercise (e.g., habituation).

Based on these investigations with animals, a progressive habituation to the performance benefits of caffeine has been proposed in humans when it is consumed chronically [14]. Previous investigations have focused on determining the changes in the performance benefits obtained with caffeine when it is consumed on a daily basis over long periods of time [15–20], but the experiments on this topic have used very diverse approaches. Several studies have used cross-sectional research protocols by comparing the improvements in performance of acute ingestion of caffeine in naïve/low caffeine consumers *vs*. individuals with habitual caffeine intake [15–17]. The outcomes of this type of research protocols are inconsistent because naïve/low caffeine consumers benefited from the acute intake of 3-to-6 mg/kg of caffeine to a similar extent compared to habitual caffeine consumers [15, 16] while a higher ergogenic effect in non-habitual caffeine consumers *vs*. habitual consumers has also been reported with 5 mg/kg of acute caffeine intake [17]. It is possible that the testing of individuals with different degrees of habituation to caffeine (e.g., low, moderate and high caffeine consumers), the use of diverse thresholds to consider naïve/low caffeine consumers (25 and 50 mg/day) and the different exercise protocols (time trial and time-to-exhaustion tests) have made it impossible to reach definitive conclusions regarding caffeine tolerance on exercise performance, as has been recently debated [16, 21].

Other studies have used longitudinal research protocols to determine the presence of tolerance to caffeine ergogenicity. Some investigations have tested the changes in the ergogenic effects obtained with acute caffeine intake (3-to-6 mg/kg) after 2–4 days of caffeine withdrawal in habitual caffeine consumers [18, 19]. These investigations have found that acute caffeine intake was equally effective before and after the caffeine withdrawal period. Thus, according to these investigations [18, 19], the absence of effects on exercise performance produced by the cessation in the intake of caffeine might be related to the lack of habituation to caffeine, although withdrawal and habituation to caffeine might follow a different time course. Stadheim et al. [22] found that caffeine ingestion of either 3 or 4.5 mg/kg increased exercise performance when it is administered over two consecutive days. Beaumont et al. [20] administered caffeine (1.5-to-3 mg/kg/day) to a group of low caffeine consumers for 28 consecutive days and they were compared to individuals that received a placebo for the same period. They found that individuals in the caffeine treatment group demonstrated a lower ergogenic effect from acute caffeine supplementation after the 28-day treatment, measured with a 30-min timed performance task, while the individuals in the control group still responded to caffeine intake after the treatment (3 mg/kg). This investigation represented the first research protocol that used consistent caffeine administration to induce a standardized caffeine habituation among individuals, but the lack of a post-supplementation comparison between caffeine and placebo trials does not allow distinguishing between the effects of tolerance and the training effects during the 28-day treatment. Lastly, with the current evidence it is impossible to determine when tolerance to the ergogenic effects of caffeine appears, but the combined data of previous investigations [20, 22] suggest that habituation to the ergogenic effect of caffeine might occur when it is ingested over 2 to 28 consecutive days.

Thus, although there is a paradigm suggesting that habitual caffeine intake may influence the performance benefits derived from acute caffeine ingestion, the scientific literature does not support this notion. The aim of the current investigation was to determine the existence and time course of tolerance to the ergogenic effects of caffeine using a longitudinal research protocol in which participants received a 20-day treatment of caffeine and placebo, to control for between-subject variability and progressive training adaptations. We hypothesized that caffeine ergogenicity would be progressively reduced when this substance is consumed day-to-day in a moderate dose (3 mg/kg/day) for 20 consecutive days but that it would still be ergogenic after a short habituation protocol.

## Materials and methods

### Ethical statement

One week before the onset of the study, the participants were fully informed of the experimental procedures and the risks and discomforts associated with the research and gave their informed written consent to participate in the investigation. The study was approved by the Camilo José Cela Research Ethics Committee and has been performed in accordance with the ethical standards as laid down in the 1964 Declaration of Helsinki and its later amendments or comparable ethical standards.

### Participants

Eleven healthy active (>4 days of training per week; > 45 min per day) individuals volunteered to participate in this investigation. They had a mean ± standard deviation (SD) age of 32.3 ± 4.9 yrs., height of 171 ± 8 cm, body mass of 66.6 ± 13.6 kg, body fat of 16.6 ± 50% and maximal oxygen uptake (VO_2_max) of 48.0 ± 3.8 mL/kg/min. There were three women in the sample who always started the treatments at the beginning of the luteal phase. Because the duration of the caffeine/placebo treatments was longer than the luteal phase of the female participants, the last days of each treatment occurred in the follicular phase. A previous investigation indicated that the pharmacokinetics of caffeine is similar in all phases of the menstrual cycle [23] and thus, it is unlikely that the change from luteal to follicular phase within the same treatment had any effect on the outcomes of this investigation. All the participants were light caffeine consumers (< 50 mg of caffeine per day), were non-smokers, who had had no previous history of cardiopulmonary diseases or musculoskeletal injuries in the previous three months. All this information was obtained from a pre-participation screening that included a medical and training history and a food frequency questionnaire. The participants were also encouraged to avoid medications or nutritional supplements for the duration of the study.

### Experimental design

**Fig 1:**
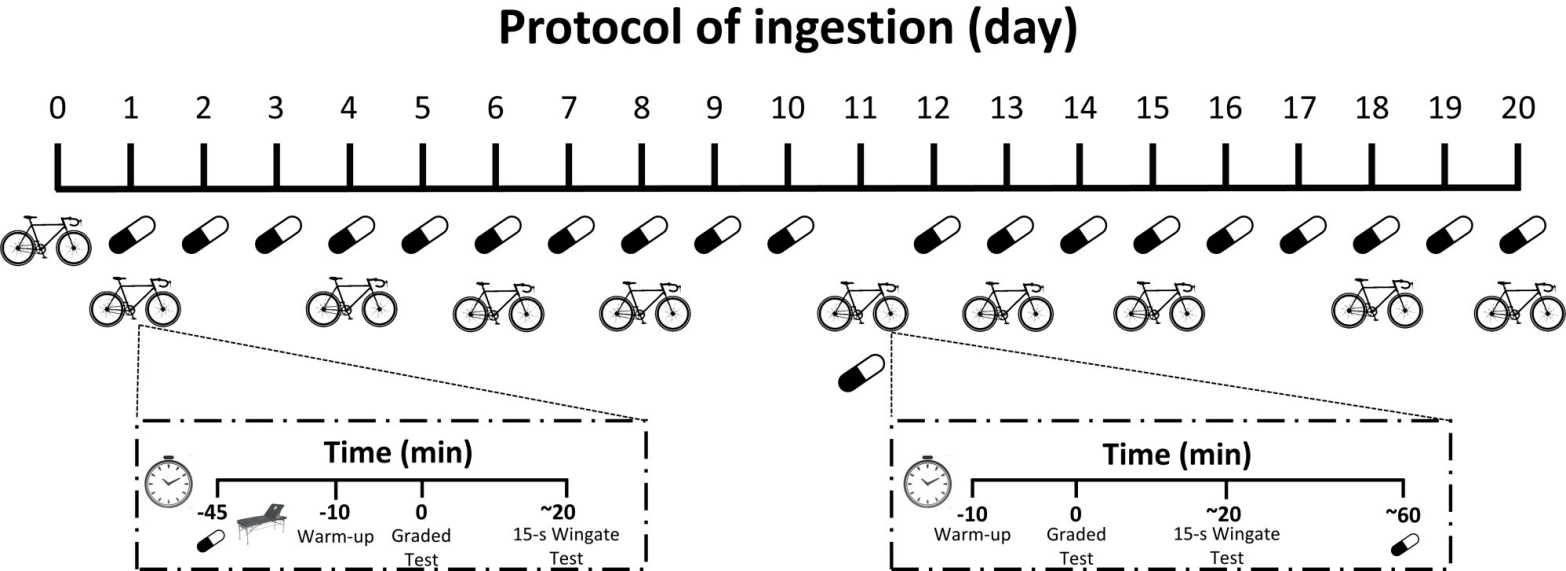
Experimental design of the investigation. Caffeine (3 mg/kg/day) or a placebo was administered for 20 consecutive days in a randomized order. Exercise performance was measured on day 0 (48 h before treatment) and on days 1, 4, 6, 8, 11, 13, 15, 18 and 20 with each protocol. Exercise performance assessment included a graded exercise test on a cycle ergometer to volitional fatigue and the 15-s Wingate test 45 min after the ingestion of the assigned capsule. Only on day 11 during each treatment, participants ingested the capsule after the end of the exercise.

A double-blind, placebo-controlled, randomized and cross-over experimental design was used in this study. Each participant took part in 2 identical protocols and thus acted as their own control: in one protocol, participants ingested an unidentifiable capsule containing 3 mg of caffeine per kg of body mass each day of the protocol (3 mg/kg/day; 100% purity, Bulk Powders, United Kingdom) for 20 consecutive days; in another protocol, they ingested the same capsule but filled with a placebo (e.g., cellulose; 100% purity, Guinama, Spain) for 20 consecutive days. The capsules were ingested daily at 9.00 am with 250 mL of tap water and in a fasted state (at least, 8 h after their last meal). Two days before the onset of each protocol of ingestion (day 0), and three times per week (i.e., day 1, 4, 6, 8, 13, 15, 18 and 20) within each treatment, participants performed the same exercise protocol composed of a maximal graded exercise test on a cycle ergometer to volitional fatigue and an adapted version of the Wingate test (all-out 15 s sprint). On day 0 of each treatment, no capsule was ingested before exercise while, on all but one of the remaining days, the exercise performance measurement always started 45 min after the ingestion of the assigned capsule (Fig 1). The experimental procedures on day 11 were different from the remaining days of measurement: on this day, the graded exercise test and the 15-s Wingate test were performed before -not after- capsule intake with the intention of having a “control” situation in the mid-point of the treatments to determine whether caffeine was still ergogenic at that time. Nevertheless, the capsule with caffeine/placebo was ingested on day 11 after the performance measurements to achieve 20 days of consecutive treatment (Fig 1). We chose to evaluate performance variables three times per week to have enough sensitivity to accurately detect habituation to the ergogenic effects of caffeine. The order of the 20-day ingestion protocols was randomized and they were separated by 7 days to allow for complete recovery and caffeine wash-out. Compliance with the treatments was verbally examined on a daily basis throughout the entire experiment and no incidences were reported during the whole experiment.

### Standardizations

For the month before the onset of the experiment, participants refrained from all sources of dietary caffeine in order to eliminate any habituation to caffeine. Consumption of dietary caffeine (coffee, tea, chocolate, sodas, energy drinks, etc) and other stimulants also ceased for the duration of the experiment and compliance was verified with dietary recalls. Participants were also encouraged to maintain their training routines and to keep a stable fitness state during the experiment. One week before the first ingestion protocol, participants underwent a routine medical screening to ensure that they were in good health and suitable for the experiment. Participants were familiarized twice with all the experimental protocols and body mass was measured (±50 g, Radwag, Poland) to calculate caffeine dosage. Environmental temperature and humidity were kept constant in all experimental trials (21.3 ± 0.3°C air temperature and 30 ± 10% relative humidity). Standardized encouragement and feedback were given to the participants in all trials by the same researcher who was blinded to the treatments. The seat and handlebar positions on the cycle ergometer were obtained in the familiarization trials and replicated for each individual in all trials.

### Experimental trials

On day 0, participants arrived at the laboratory at 8.00 and body fat percentage was measured by bioelectrical impedance (B-418, Tanita, Japan). Participants then dressed in a T-shirt, and shorts and a heart rate belt (Wearlink, Polar, Finland) was attached to their chest. After that, they rested supine for 45 min. Resting heart rate and systolic and diastolic blood pressure (M6 Comfort, Omron, Japan; by triplicate) were measured during the last 5 min of the resting period.

After the resting measurements, participants performed a 10-min standardized warm-up on a cycle ergometer (SNT Medical, Cardgirus, Spain) at 50 W and then exercise intensity was progressively increased by 25 W/min (stepwise increases) until volitional fatigue. Pedaling frequency was individually-chosen (between 75 and 90 rpm) but maintained during the whole graded exercise test and replicated in all experimental trials. During the test, oxygen uptake (VO_2_) was continuously measured by means of a breath-by-breath analyzer (Metalyzer 3B, Cortex, Germany) and the data were averaged every 15 s. In this graded exercise test, the wattage attained at exhaustion (Wmax; aerobic peak power) and the VO_2_max were determined. Wmax was recorded as the exercise load on the cycle ergometer at the moment that participants abruptly stopped pedaling or when an individual’s pedaling frequency was lower than 50 rpm. VO_2max_ was defined as the highest VO_2_ value obtained during the test. The VO_2max_ was considered valid when participants rated their perceived exertion higher than 19 on the Borg scale, the VO_2_ difference between the last two consecutive workloads was less than 0.10 L/min^-1^, respiratory exchange ratio was higher than 1.10, heart rate was higher than 80% of the age-adjusted estimate of maximal heart rate and lactate concentration was more than 7 mmol/L [24]. Certified calibration gases (16.0% O_2_; 5.0% CO_2_, Cortex, Germany) and a 3-L syringe were used to calibrate the gas analyzer and the flow meter before each trial. One minute after the end of the graded test, a blood sample was obtained from a fingertip to analyze blood lactate concentration (Lactate Pro 2, Arkay, Japan).

After the maximal graded exercise test, participants continued pedaling at 50 W for 7 min. After this, they performed a 15-s Wingate test on the same cycle ergometer (SNT Medical, Cardgirus, Spain) and with a load that represented 7.5% of body mass, as previously described [25]. For this test, participants started from a stationary position with their dominant leg ready to pedal and they were told that “they had to pedal as fast as they could from the beginning and for the whole duration of the test”. After the “start” command, the resistance load was progressively applied within 3 s to produce an acceleration phase, as previously suggested [26]. The researcher verified that participants remained seated during the whole test. During the Wingate test, cycling power was obtained with a frequency of 1 Hz and the peak and mean cycling power obtained during the test were recorded. One minute after the end of the test, blood lactate concentration was measured as previously described.

Participants were asked about their self-perceived endurance and exertion just after the graded exercise test and about their self-perception of muscle power just after the 15-s Wingate test. This questionnaire included a 1–10 point scale to assess each item, and participants were previously informed that 1 point meant a minimal amount of that item and 10 points meant a maximal amount of the item. The questionnaire has been previously used to assess perceived ergogenicity after the intake of caffeine [27].

The protocol described above for day 0 was replicated for each day of measurement during the 20-day treatment (e.g., days 1, 4, 6, 8, 13, 15, 18 and 20) but with the administration of caffeine or placebo 45 min before the onset of the performance tests. On day 11, participants ingested the assigned capsule after the end of the exercise testing (Fig 1) to produce a continuous 20-day ingestion protocol. Seven days after the conclusion of the first protocol of ingestion (placebo or caffeine), the remaining protocol of ingestion was carried out mimicking the procedures described above.

### Statistical analysis

Data were collected as previously indicated and the results of each test were subsequently blindly introduced into the statistical package SPSS v 20.0 for later analysis. Normality was tested for each variable with the Shapiro-Wilk test. All the variables included in this investigation presented a normal distribution (*P* > 0.05) and parametric statistics were used to determine the ergogenicity of caffeine. Differences between the caffeine *vs*. placebo protocols were determined by two-way analysis of variance (substance × day of ingestion) with repeated measures. After a significant *F* test (Geisser-Greenhouse correction for the assumption of sphericity), differences between means were identified using Tukey’s HSD *post hoc*. The significance level was set at *P* < 0.05.

The effect size was also calculated in all pairwise comparisons to allow a magnitude-based inference approach [28]. Specifically, the effect-size statistic ± 90% confidence intervals (CI) was used on log transformed data to reduce bias due to non-uniformity of error. The smallest significant standardized effect threshold was set as 0.2, and a qualitative descriptor was included to represent the likelihood of exceeding this threshold. Ranges of likelihood <1% indicated almost certainly no chances of change; 1% to 5%, very unlikely; 5% to 25%, unlikely; 25% to 75%, possible; 75% to 95%, likely; 95% to 99%, very likely; >99%, most likely. Differences were rated as unclear when likelihood exceeded >5% in both positive/negative directions. Effect sizes were interpreted according to the following ranges: <0.2, trivial; 0.2–0.6, small; 0.6–1.2, moderate; 1.2–2.0, large; 2.0–4.0, very large and; >4.0, extremely large [28].

## Results

### Incremental exercise testing

**Fig 2:**
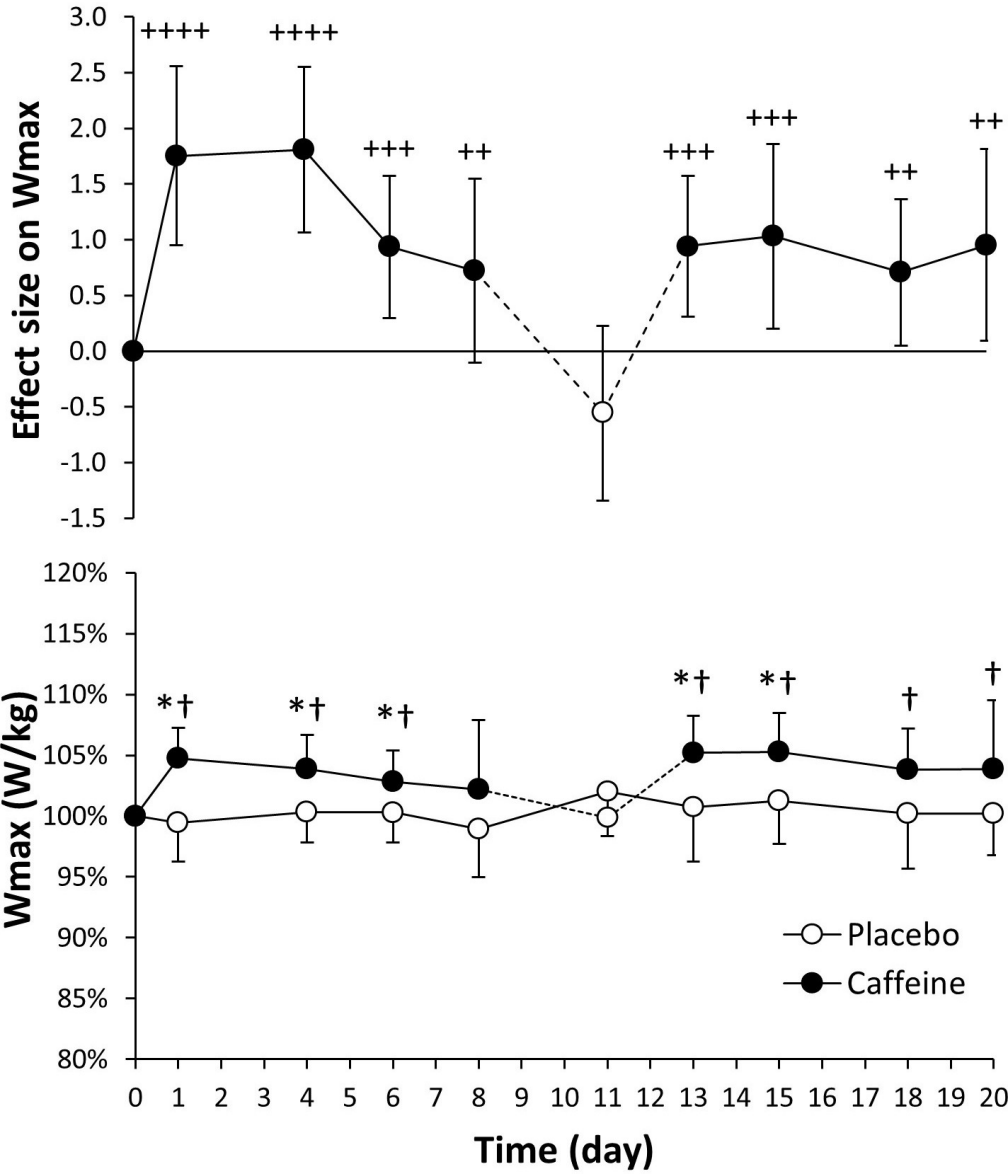
Peak cycling power (W_max_) obtained during a graded exercise test with the administration of 3 mg/kg/day of caffeine or a placebo for 20 consecutive days. The upper panel depicts the effect size (± 90% confidence intervals) for all pairwise comparisons. Only effect sizes with a possible likelihood of difference (>25%) are categorized: (++++) most likely, (+++) very likely, (++) likely, (+) possibly. The lower panel depicts data presented as mean ± standard deviation. The data have been normalized with respect to the values obtained on day 0 of each treatment to provide a better comparison of the caffeine ergogenic effect in the studied variables. (*) Caffeine different from placebo for the same day, *P* < 0.05. (†) Different from day 0 within the same treatment, *P* < 0.05.

The values for Wmax were very comparable for day 0 in placebo and caffeine ingestion protocols (3.99 ± 0.75 and 3.97 ± 0.75 W/kg, respectively; *P* = 0.66). In comparison to the placebo, the daily ingestion of caffeine increased Wmax for the trials performed within the first 18 days of the caffeine protocol (Fig 2, lower panel; *P* < 0.05), except for day 11 when caffeine and placebo were ingested after the testing. Besides, daily caffeine intake also increased Wmax for the whole treatment with respect to day 0 of the caffeine protocol (*P* < 0.05). In contrast, Wmax remained constant in the placebo protocol. In the pairwise comparisons, the effect size of caffeine intake on Wmax was large for day 1 and day 4 and decreased to moderate afterwards (Fig 2, upper panel).

**Fig 3:**
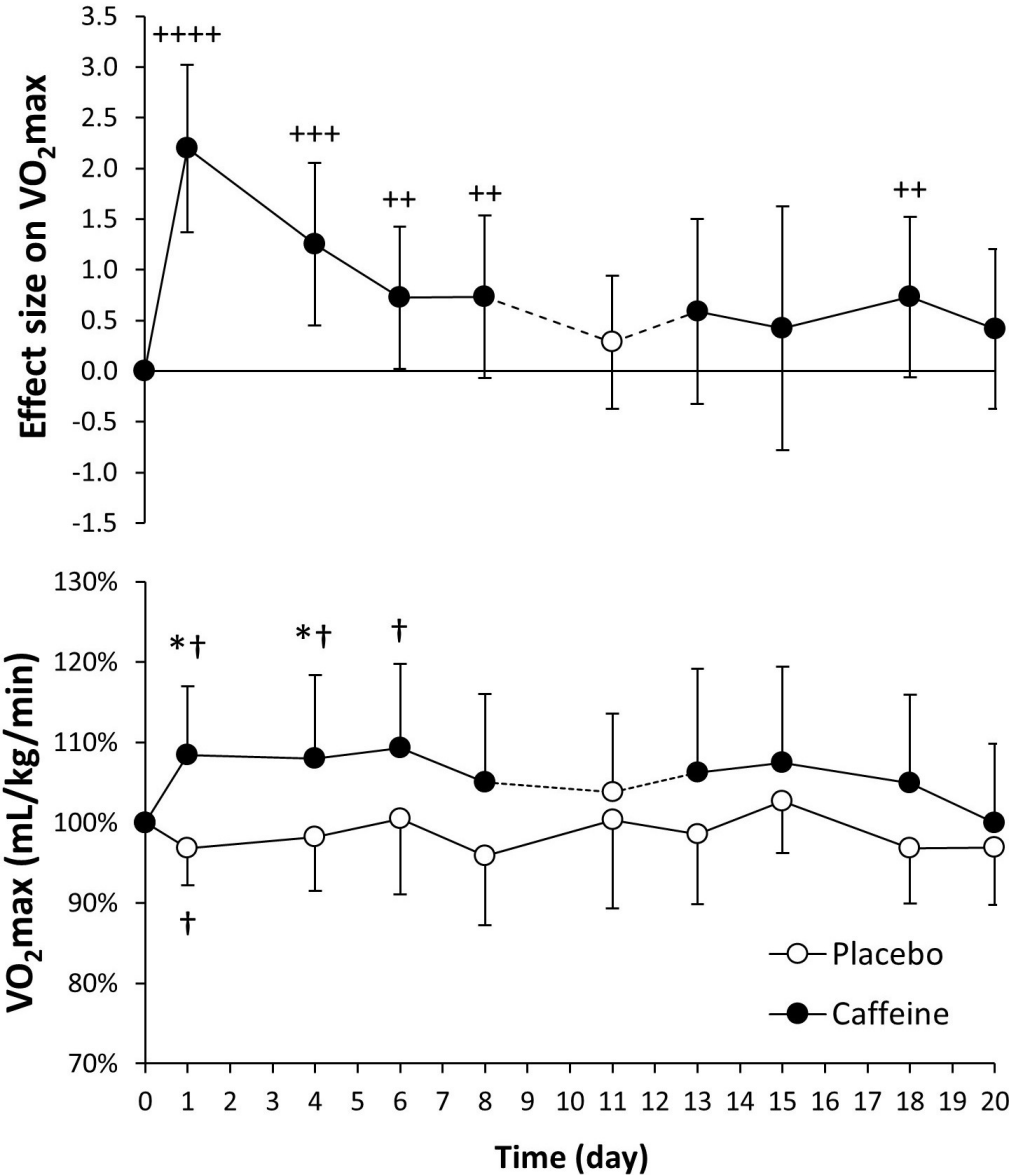
Maximal oxygen uptake (VO_2_max) obtained during a graded exercise test with the administration of 3 mg/kg/day of caffeine or a placebo for 20 consecutive days. The upper panel depicts the effect size (± 90% confidence intervals) for all pairwise comparisons. Only effect sizes with a possible likelihood of difference (>25%) are categorized: (++++) most likely, (+++) very likely, (++) likely, (+) possibly. The lower panel depicts data presented as mean ± standard deviation. The data have been normalized with respect to the values obtained on day 0 of each treatment to provide a better comparison of the caffeine ergogenic effect in the studied variables. (*) Caffeine different from placebo for the same day, *P* < 0.05. (†) Different from day 0 within the same treatment, *P* < 0.05.

The values for VO_2_max were very comparable for day 0 in placebo and caffeine ingestion protocols (47.9 ± 8.0 and 46.1 ± 8.4 mL/kg/min; *P* = 0.19). In the day-to-day comparison to the placebo, the ingestion of caffeine increased VO_2_max on days 1 and 4 (Fig 3, lower panel; *P* < 0.05), and on days 1, 4 and 6 with respect to day 0 of the caffeine protocol (*P* < 0.05). VO_2_max remained constant in the placebo protocol, except for a reduction on day 1 with respect to day 0 (*P* < 0.05). In the pairwise comparisons, the effect size of caffeine intake on VO_2_max was very large for day 1, large for day 4 and from moderate to small afterwards (Fig 3, upper panel).

The ingestion of caffeine increased maximal heart rate on day 1 and 8 respect to the placebo (Table 1) but there was no other statistically significant difference between caffeine vs. placebo in the remaining trials. In the pairwise comparisons, the effect size of caffeine intake on maximal heart rate was small for the first 8 days of ingestion and tended to be insignificant afterwards. The ingestion of caffeine increased maximal ventilation on days 1 and 4 respect to the placebo (Table 1) but did not produce any other statistically significant difference for caffeine vs. placebo comparison afterwards. The effect size of caffeine on maximal ventilation was small on days 1 and 4 and was then reduced afterwards.

**Table 1:** Maximal heart rate (beats/min) and maximal pulmonary ventilation (L/min) during a maximal graded cycling test with the administration of 3 mg/kg/day of caffeine or a placebo for 20 consecutive days.

Maximal heart rate (bpm)					Maximal ventilation (L/min)			
Day	Placebo	Caffeine	Effect size (±90% CI)	Qualitative inference	Placebo	Caffeine	Effect size (±90% CI)	Qualitative inference
0	177±12	178±13	0.1(-0.2–0.3)	Likely trivial	130±23	136±33	0.2(-0.1–0.50)	Possibly positive
1	175±11	181±12*	0.4(0.1–0.7)	Likely positive	131±26	146 ±36*	0.5(0.1–0.8)	Likely positive
4	176±10	179±8	0.3(0–0.6)	Possibly positive	134±24	148±34*†	0.5(0.2–0.7)	Likely positive
6	178±11	180±11	0.2(-0.2–0.5)	Possibly positive	138±32	142±37	0.1(-0.2–0.4)	Unclear
8	174±10	180±11*	0.5(0.2–0.7)	Likely positive	136±32	141±36	0.1(-0.1–0.4)	Possibly trivial
11	177±9	172±10†	-0.5(-0.9–0)	Likely negative	134±29	133±29	-0.0(-0.2–0.2)	Likely trivial
13	177±10	177±12	0.1(-0.3–0.5)	Unclear	135±26	147±36	0.4(0.1–0.7)	Likely positive
15	175±13	179±8	0.2(-0.2–0.6)	Possibly positive	139±30	150±33†	0.3(0.1–0.6)	Likely positive
18	176± 10	177±10	0.1(-0.2–0.4)	Unclear	136±31	143±33	-0.2(-0.1–0.5)	Possibly positive
20	176±10	178±9	0.2(-0.1–0.5)	Possibly positive	136±30	145±28†	0.3(0.1–0.6)	Likely positive

CI = confidence interval;

(*) Caffeine different from placebo for the same day, *P* < 0.05.

(†) Different from day “0” within the same treatment, *P* < 0.05.

Note: On days “0” and “11”, both the placebo and caffeine trials were performed without the administration of any capsule.

Blood lactate concentration after the graded exercise test on day 1, and 15 (Table 2; *P* < 0.05) was higher with the ingestion of caffeine in comparison to the placebo and the effect size on these days was moderate. Participants perceived a higher endurance capacity in the incremental exercise test with caffeine vs. placebo on days 1 and 13 (Table 3; *P* < 0.05) while the size of the caffeine effect was small on these same days. Besides, on days 1, 13, 15 and 18 there was a significant increase in perceived endurance capacity with respect to day 0 of the caffeine protocol (*P* < 0.05). There were no effects of caffeine on perceived exertion after the incremental exercise test, which was always perceived as > 9 points on the 1–10 point scale (Table 3).

**Table 2:** Blood lactate concentration (mmol/L) after a maximal graded cycling test (VO_2max_) and after a 15-s Wingate test (Wingate) with the administration of 3 mg/kg/day of caffeine or a placebo for 20 consecutive days.

Graded exercise test					15-s Wingate test			
Day	Placebo	Caffeine	Effect size (±90% CI)	Qualitative inference	Placebo	Caffeine	Effect size (±90% CI)	Qualitative inference
0	13.1±2.8	13.3±2.9	0.1(-0.5–0.6)	Unclear	13.4±3.7	12.9±2.6	-0.0(-0.8–0.7)	Unclear
1	11.8±3.3	14.0±2.8*	0.6(0.1–1.1)	Likely positive	13.3±4.5	14.8±2.5†	0.4(-0.1–0.9)	Possibly positive
4	12.8±2.7	13.6±2.1	-0.1(-0.5–0.4)	Unclear	12.2±3.6	14.5±2.8†	0.6(-0.2–1.6)	Likely positive
6	13.8±3.2	13.8±4.5	0.0(-0.7–0.6)	Unclear	12.3±3.5	14.0±4.5	0.4(-0.2–0.9)	Possibly positive
8	12.7±3.2	13.1±2.4	0.1(-0.5–0.8)	Unclear	12.3±4.3	14.9±4.0	0.5(-0.1–1.1)	Likely positive
11	13.8±2.0	13.5±3.9	-0.4(-1.4–0.6)	Unclear	13.3±3.7	13.0±3.4	0.1(-0.3–0.5)	Unclear
13	12.6±3.8	14.5±3.6	0.6(0.1–1.1)	Likely positive	11.9±2.8	13.9±4.8†	1.1(0.1–2.1)	Likely positive
15	12.3±2.0	14.8±4.0*	0.9(0.0–1.9)	Likely positive	11.8±3.2	14.0±1.8†	1.0(0.4–1.7)	Very likely positive
18	12.9±3.4	15.0±4.8	-0.5(-0.3–1.3)	Unclear	13.8±4.6	13.4±4.2	-0.1(-0.9–0.7)	Unclear
20	13.3±3.5	12.8±3.1	-0.1(-0.8–0.6)	Unclear	14.0±4.4	13.9±2.0	0.1(-0.3–0.6)	Unclear

CI = confidence interval;

(*) Caffeine different from placebo for the same day, *P* < 0.05.

(†) Different from day “0” within the same treatment, *P* < 0.05.

Note: On days “0” and “11”, both the placebo and caffeine trials were performed without the administration of any capsule

**Table 3:** Subjective feelings of endurance and exertion (1–10 point scales) after a graded exercise test, and perceived muscle power after a 15-s Wingate test with the administration of 3 mg/kg/day of caffeine or a placebo for 20 consecutive days.

	Perceived endurance				Perceived exertion				Perceived muscle power			
Day	Placebo	Caffeine	Effect size (±90% CI)	Qualitative inference	Placebo	Caffeine	Effect size (±90% CI)	Qualitative inference	Placebo	Caffeine	Effect size (±90% CI)	Qualitative inference
0	5.6±1.2	5.4±0.9	-0.1(-0.6–0.4)	Unclear	9.2±1.0	9.5±0.6	0.3(0–0.6)	Likely positive	5.4±1.0	5.8±0.7	0.4(-0.1–0.9)	Possibly positive
1	5.3±1.5	6.1±0.9*†	0.5(0.1–1.0)	Likely positive	9.4±1.1	9.4±0.8	-0.1(-0.4–0.3)	Unclear	5.7±1.6	6.4±0.7†	0.3(-0.2–0.8)	Possibly positive
4	5.7±1.1	5.9±1.0	0.2(-0.3–0.8)	Unclear	9.4±0.8	9.5±0.7	0.1(-0.5–0.7)	Unclear	6.0±0.7	6.2±1.1	0.0(-0.8–0.9)	Unclear
6	5.9±0.9	5.8±0.9	-0.1(-0.4–0.3)	Unclear	9.5±0.7	9.6±0.7	0.1(-0.1–0.3)	Unlikely positive	6.3±0.6	5.7±1.3	-1.0(-2.2–0.2)	Unclear
8	5.3±1.5	5.5±1.8	0.1(-0.5–0.7)	Unclear	9.5±0.8	9.6±0.5	0.1(-0.3–0.5)	Possibly negative	5.0±1.5	5.1±1.7	0.1(-0.5–0.7)	Unclear
11	6.1±1.0	5.0±1.8	-1.3(-3.0–0.3)	Unclear	9.5±1.0	9.3±1.0	-0.2(-1-0.6)	Unclear	6.3±1.3	5.0±1.7	-1.0(-2.3–0.2)	Unclear
13	5.5±1.4	6.3±0.8†*	0.5(0.1–0.9)	Likely positive	9.7±0.5	9.6±0.7	-0.2(-1.0–0.6)	Unclear	6.2±1.2	6.2±1.5	-0.1(-0.9–0.7)	Unclear
15	5.8±1.5	6.2±0.9†	0.2(0.3–0.8)	Unclear	9.6±0.7	9.7±0.5	0.0(-0.6–0.6)	Unclear	5.8±1.2	5.8±1.4	0.0(-0.8–0.8)	Unclear
18	5.8±1.0	6.1±0.9†	0.3(-0.2–0.8)	Possibly positive	9.8±0.4	9.6±0.9	0.0(-0.4–0.3)	Unclear	5.9±1.2	6.1±1.1	0.2(-0.4–0.7)	Unclear
20	5.8±1.2	5.6±1.6	-0.1(-0.7–0.4)	Unclear	9.8±0.4	9.6±0.9	-0.2(1.5–1.0)	Unclear	5.7±0.0	6.3±0.9	0.6(-0.0–1.2)	Likely positive

Note: All variables were assessed with 1–10 point scales where 1 point meant minimal amount of the variable and 10 points meant maximal amount of that variable (5 points was the value used to indicate not feeling any difference from a regular day). CI = confidence interval;

(*) Caffeine different from placebo for the same day, *P* < 0.05.

(†) Different from day “0” within the same treatment, *P* < 0.05.

Note: On days “0” and “11”, both the placebo and caffeine trials were performed without the administration of any capsule.

### 15-s Wingate test

**Fig 4:**
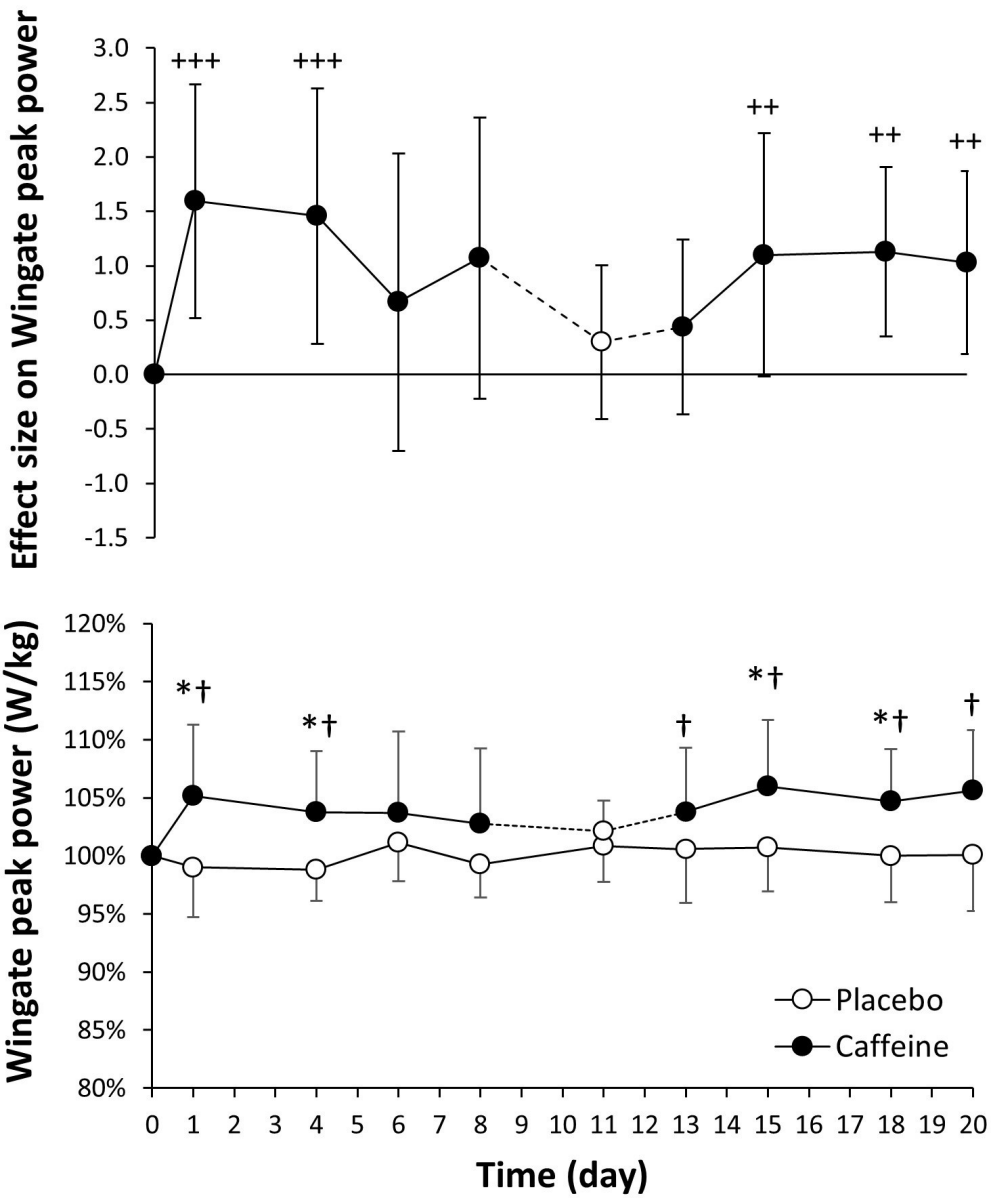
Peak cycling power obtained during an adapted version of the Wingate test (all-out 15 s sprint) with the administration of 3 mg/kg/day of caffeine or a placebo for 20 consecutive days. The upper panel depicts the effect size (± 90% confidence intervals) for all pairwise comparisons. Only effect sizes with a possible likelihood of difference (>25%) are categorized: (++++) most likely, (+++) very likely, (++) likely, (+) possibly. The lower panel depicts data presented as mean ± standard deviation. The data have been normalized with respect to the values obtained on day 0 of each treatment to provide a better comparison of the caffeine ergogenic effect in the studied variables. (*) Caffeine different from placebo for the same day, *P* < 0.05. (†) Different from day 0 within the same treatment, *P* < 0.05.

**Fig 5:**
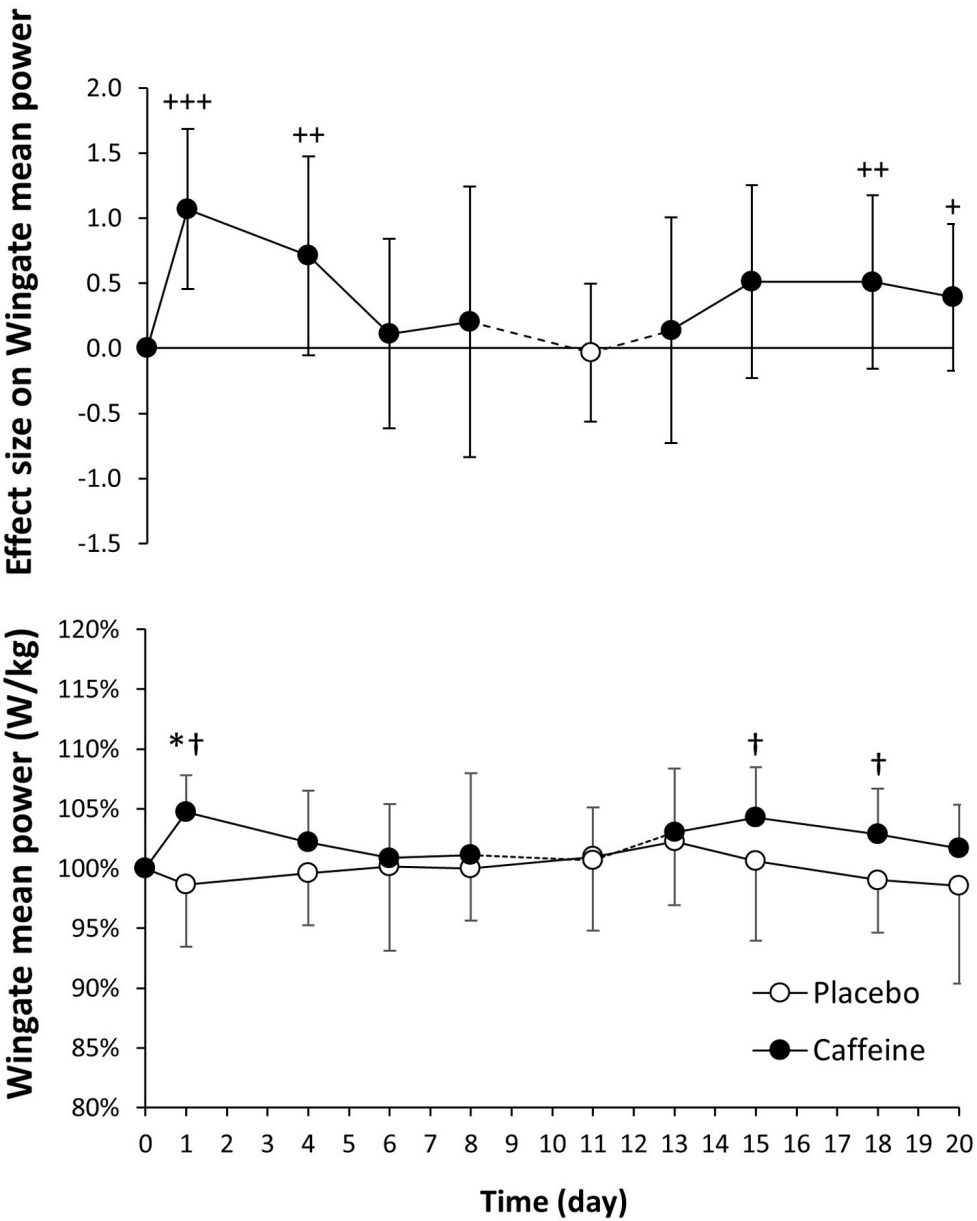
Mean cycling power obtained during an adapted version of the Wingate test (all-out 15 s sprint) with the administration of 3 mg/kg/day of caffeine or a placebo for 20 consecutive days. The upper panel depicts the effect size (± 90% confidence intervals) for all pairwise comparisons. Only effect sizes with a possible likelihood of difference (>25%) are categorized: (++++) most likely, (+++) very likely, (++) likely, (+) possibly. The lower panel depicts data presented as mean ± standard deviation. The data have been normalized with respect to the values obtained on day 0 of each treatment to provide a better comparison of the caffeine ergogenic effect in the studied variables. (*) Caffeine different from placebo for the same day, *P* < 0.05. (†) Different from day 0 within the same treatment, *P* < 0.05.

The values for the Wingate peak power were very comparable for day 0 in placebo and caffeine ingestion protocols (9.48 ± 0.44 and 9.35 ± 0.54 W/kg; *P* = 0.39). In comparison to the placebo, the daily intake of caffeine increased Wingate peak power on days 1, 4, 15 and 18 (Fig 4, lower panel; *P* < 0.05). Caffeine also increased Wingate peak power on days 1, 4, 13, 15, 18 and 20 with respect to day 0 of the caffeine protocol (*P* < 0.05). The effect size of caffeine intake on Wingate peak power was large on days 1 and 4 and was reduced to moderate/small afterwards (Fig 4, upper panel). From similar values on day 0 (8.22 ± 0.46 and 8.15 ± 0.43 W/kg; *P* = 0.57) Wingate mean cycling power only increased with caffeine on day 1 of ingestion with respect to the placebo (*P* < 0.05), and on days 1, 15 and 18 with respect to day 0 of the caffeine protocol (Fig 5, lower panel). The effect size of caffeine intake on Wingate mean power was moderate on days 1 and 4 and decreased to small afterwards (Fig 5, upper panel). Both peak and mean cycling power remained stable in the placebo protocol.

There were no statistically significant differences in blood lactate concentration after the Wingate test in caffeine vs. placebo protocols although caffeine produced a higher blood lactate concentration on days 1, 4, 13 and 15 with respect to day 0 of the caffeine ingestion protocol (Table 2; *P* < 0.05). On these same days, the effect size of caffeine ingestion over the placebo was small-to-moderate. There were no statistically significant differences in perceived muscle power during the Wingate test in caffeine vs. placebo with small effect sizes in pairwise comparisons. However, caffeine produced a higher perceived muscle power on day 1, with respect to day 0 of the caffeine protocol (Table 3; *P* < 0.05).

## Discussion

The aim of the study was to determine the existence of tolerance to the ergogenic effects of caffeine using a cross-over, repeated measures longitudinal research protocol that included 20 days of consecutive administration of a moderate dose of caffeine (3 mg/kg) or a placebo. The similar baseline values in all performance variables for the caffeine and placebo protocols, together with the absence of training/familiarization effects within the placebo treatment (as the stable values in all performance variables indicate) present the following main outcomes: a) in comparison to the ingestion of a placebo, for 15 days of consecutive ingestion, caffeine increased peak cycling power obtained during a maximal graded exercise test and VO_2_max for 4 days (Figs 2 and 3); b) the ingestion of caffeine also increased for ~18 days of consecutive ingestion the maximal power obtained in a 15-s all-out cycling test, although the ergogenic effect on mean cycling power was only evident after the first day of caffeine ingestion (Fig 4); c) the analysis of the effect sizes derived from the pairwise comparison between the ingestion of 3 mg/kg/day of caffeine vs. placebo revealed that there is a gradual tolerance to the ergogenic effects of caffeine because the size of its ergogenic effect peaked on day 1 of ingestion and decreased afterwards. However, it is necessary to mention that caffeine still exerted small-to-moderate improvements on physical performance after 20 days of consecutive administration (Figs 2–5). All these results suggest that there is a relative reduction in the ergogenic effects of caffeine when a moderate dose of this substance is ingested daily, but it still has the capacity to improve performance after 20 days of consecutive ingestion.

One of the mechanism that explains the increase in physical performance with acute administration of caffeine is related to its ability to act as an adenosine A_1_ and A_2A_ receptor antagonist [6, 7]. Specifically, caffeine is structurally similar to adenosine, a neuromodulator that might induce central fatigue through reduced neuroexcitability and decreased release of noradrenaline and dopamine, among other neurotransmitters [29]. In this regard, the micromolar tissue concentrations of caffeine resulting from ingestion of low to moderate doses can block A_1_ and A_2A_ adenosine receptors [30]. Thus, the intake of caffeine would blunt the fatiguing effects of adenosine through inhibition of the decreased release of the above-mentioned neurotransmitters. This theory is well-supported because it has been found that caffeine infusion (up to 4.3 mg/kg) can block up to 50% of adenosine receptors in the human brain [8]. However, the concept of tolerance/habituation to the ergogenic effects of caffeine has been based on previous research with rodents that reported the capacity of caffeine to block adenosine A_1_ and A_2A_ receptors and the upregulation in the number of adenosine receptors in neural and vascular tissues of the brain with the regular consumption of caffeine [31, 32]. More recent investigations have shown that tolerance to caffeine might be driven by alterations in gene expression in striatum while the changes in caffeine metabolism induced after long-term caffeine intake cannot explain development of tolerance to caffeine [33]. All this information suggests that daily caffeine intake results in a higher likelihood of adenosine binding the newly created adenosine receptors, progressively reducing the adenosine-blocking-action of caffeine, coupled with adaptive changes in gene expression that ultimately lead to development of locomotor tolerance to caffeine.

However, the evidence for tolerance to the stimulatory effects of caffeine in humans is inconclusive. In variables of cognitive performance, caffeine administration equally improved performance in habitual and non-consumers of caffeine [34] or even a higher performance benefit was found in high-caffeine consumers than in moderate caffeine consumers [35]. Several investigations indicated that the efficacy of caffeine to improve physical performance may be reduced in individuals who consume moderate to high doses of caffeine daily (130–300 mg/day) in comparison to low caffeine consumers (40–50 mg/day; [17, 36]). Furthermore a progressive tolerance to the ergogenic effects has also been found in individuals that consume 1.5–3.0 mg/kg/day of caffeine for 28 consecutive days [20]. Nevertheless, other investigations have determined that habitual caffeine consumers might also benefit from the ergogenic effects of caffeine even when their self-reported daily caffeine intake surpassed 300 mg/day [15, 16, 18, 37]. It is likely that the differences in the research protocols chosen to study this question, specifically, the use of individuals with different daily intakes and periods of habituation to caffeine, have contributed to the different outcomes. However, previous investigations suggest that caffeine can be ergogenic even after habituation to it.

The experimental design used in the current investigation is innovative, respect to previous research, because the same individuals were tested in two identical treatments with caffeine and a placebo and they acted as their own controls during the whole experiment. All the participants were low caffeine consumers, withdrew from caffeine for one month before the experiment and received the same daily dosage per kg of body mass of caffeine to normalize the protocol for habituation to caffeine. The effects of caffeine intake on performance were measured three times per week to improve accuracy in identifying tolerance to it. Besides, we used a maximal graded exercise test and the Wingate test to broaden the range of the investigation to endurance-like and power-like performance. Finally, in addition to the traditional statistical analysis based on p values, we used a magnitude-inference approach to aid in the identification of tolerance to caffeine. With this experimental design, it was possible to determine a progressive tolerance to the ergogenic effects of caffeine, present in both maximal/peak values of aerobic-based and power-based exercise tests because of the progressive reduction in the effect size of caffeine ergogenicity. However, the results of the current investigation dispute the existence of a complete tolerance to caffeine after 20 days of continuous ingestion because the size of the caffeine ergogenic effect was moderate/small even after this period of consecutive ingestion (Figs 2–5). In the light of the current results, we can conclude that caffeine benefits physical performance after 20 days of consecutive ingestion although the magnitude of the ergogenic effect is somewhat reduced when compared to the first day of ingestion (i.e., non-habituation to caffeine intake). In the current investigation, the dose of caffeine administrated (3 mg/kg/day, equivalent to ~200 mg/day in our participants) was moderate and it was below the daily ingestion reported by habitual caffeine consumers [15, 16, 18, 37]. Thus, it should be investigated whether a higher dose of caffeine intake might change the time course of tolerance to the performance benefits of caffeine.

We want to highlight day 11 of ingestion because participants performed the testing protocols before -instead of after- the ingestion of the assigned capsule, for both caffeine and placebo treatments. This particular variation in the day-to-day protocol of caffeine intake was chosen to assess caffeine tolerance at the mid-point of the treatments but did not interrupt the day-to-day ingestion of the substances. On day 11, caffeine Wmax and peak power in the Wingate decreased to a similar value to the placebo trial, while these performance variables again increased on day 13 with the administration of caffeine, confirming that it was still effective to increase performance and thus, no tolerance had been produced at this time. In the placebo ingestion protocol, all the performance variables were maintained similar to day 0 during the 20 days of treatment, suggesting a stable fitness level for the duration of this protocol. In contrast, caffeine produced numerous differences not only in the pairwise comparison to the placebo treatment but also to day 0 of the caffeine ingestion protocol. These outcomes reinforce the notion of the maintained ergogenicity of caffeine during a 20-day period together with a progressive reduction in the extent of its ergogenic effect.

After the testing, the participants were asked about their feelings of exertion, endurance and muscle power to determine the perceived ergogenicity of the treatments (Table 3). They perceived high exertion after the graded exercise test on all days of the treatments and in both ingestion protocols, as was expected for this maximal test. They also perceived a significantly higher endurance capacity the first day of caffeine ingestion and after 13 days of ingestion which do not coincide with the increases of caffeine on Wmax (Fig 2). Similarly, the perception of muscle power was not significantly higher with caffeine in comparison to the placebo on any of the days of treatment, despite caffeine increasing peak cycling power on several occasions (Fig 4). These data indicate that caffeine can be ergogenic when ingested daily for 20 consecutive days even when the participants did not feel they had given an enhanced performance after the ingestion of this stimulant.

The experimental design used in the present investigation had several limitations that should be discussed for a correct application of the results. First, the duration of the chronic caffeine intake lasted for 20 days and it is impossible to determine if greater tolerance to the ergogenic effects of caffeine would occur after 20 days of ingestion. In addition, individuals considered as habitual caffeine consumers can use this substance daily for years and thus, this investigation might not be representative of this chronic consumption [16]. Second, we used laboratory testing with a high reproducibility (between-day coefficient of variation for Wmax = 2.4 ± 1.1%; for peak cycling power in the 15-s Wingate = 2.6 ± 0.9%) to determine loss of responsiveness to the ergogenic effects of caffeine. However, these results should be confirmed with further research protocols that use field testing or performance assessments in real or simulated sport competition to accurately convey the conclusions of this investigation to athletes in sport situations. Third, tolerance to the ergogenic effects of caffeine was tested with a moderate dose (e.g., 3 mg/kg/day) because we have found that this dose is effective to increase performance in a myriad of laboratory-based tests and sports [38–43]. Nevertheless, it is likely that lower or higher doses of caffeine produce a different time course for tolerance to the ergogenic effect of this substance. Fourth, the obtaining of maximal/peak values in the performance tests used required high levels of motivation. To maintain motivation in all tests throughout the experiment, a researcher, blinded to the treatments, gave standardized encouragement commands in each trial but it is still possible that the changes in motivation within each treatment affected the outcomes of the investigation. Finally, we did not obtain blood samples during the ingestion protocols to determine plasma caffeine concentration before each experimental trial and thus, we cannot determine whether the changes in caffeine metabolism are in part responsible for the tolerance to the ergogenic effect of caffeine. Despite these limitations, the results of this investigation can shed light on the changes in caffeine ergogenicity when it is consumed daily in a moderate dose.

In summary, the daily intake of caffeine (3 mg/kg/day) significantly increased peak cycling power during a maximal incremental test for the first 15 days of ingestion and improved VO_2_max for the first 4 days, when compared to the same treatment with a placebo. Day-to-day pre-exercise caffeine intake also produced higher peak cycling power during the 15-s Wingate tests for ~18 days of intake, although Wingate mean power was only increased on the first day of ingestion with respect to the placebo. After these periods of time, the increases in performance of these variables with caffeine were not statistically different from the placebo. These results suggest the existence of progressive tolerance to the performance benefits of caffeine, particularly because the magnitude of the ergogenic effect of caffeine was higher on the first day of ingestion for both aerobic-based and power-based exercise and decreased afterwards. Nevertheless, the effect size of the caffeine-placebo pairwise comparison remained small-to-moderate after 20 days of consecutive ingestion which suggests that caffeine was still ergogenic after this period of time.

## Supporting information

S1 Fig Individual responses for the percentage of change produced by the ingestion of caffeine over a placebo on peak cycling power (W_max_; upper panel) and maximal oxygen uptake (VO_2_max; lower panel) when 3 mg/kg/day of caffeine were administrated for 20 consecutive days. Each dot represents the caffeine vs placebo change in each individual and positive values indicate a superior performance with caffeine over placebo.
(TIF)

S2 Fig Individual responses for the percentage of change produced by the ingestion of caffeine over a placebo on Wingate peak (upper panel) and mean cycling power (lower panel) when 3 mg/kg/day of caffeine were administrated for 20 consecutive days. Each dot represents the caffeine vs placebo change in each individual and positive values indicate a superior performance with caffeine over placebo.
(TIF)
