# Supplementary figures and images for: Time course of tolerance to the performance benefits of caffeine
Source: PLoS One. 2019 Jan 23;14(1):e0210275. doi: 10.1371/journal.pone.0210275 (PMC6343867; doi:10.1371/journal.pone.0210275)

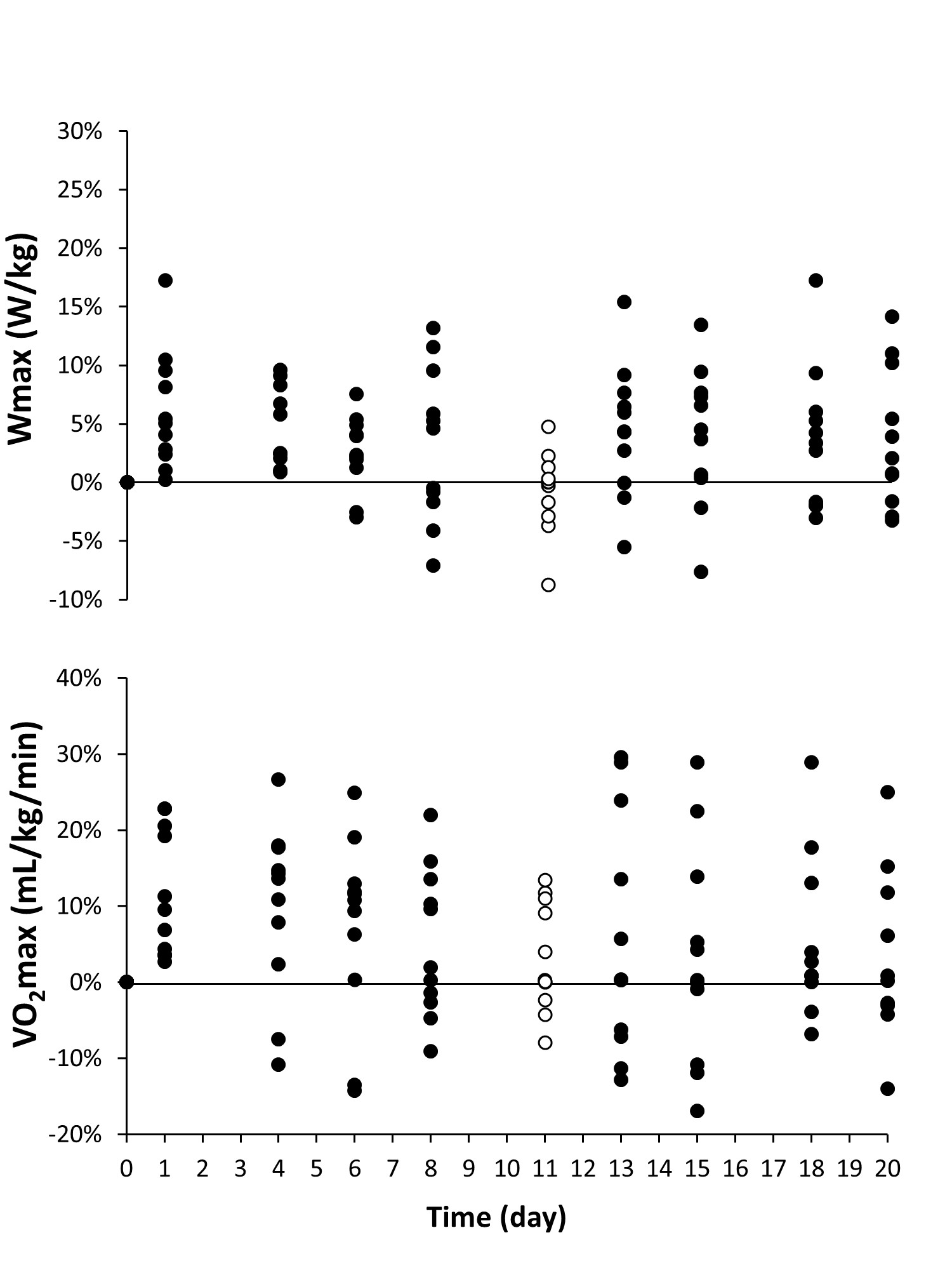

Supplement: S1 Fig — Each dot represents the caffeine vs placebo change in each individual and positive values indicate a superior performance with caffeine over placebo. (TIF) [file pone.0210275.s001.tif]

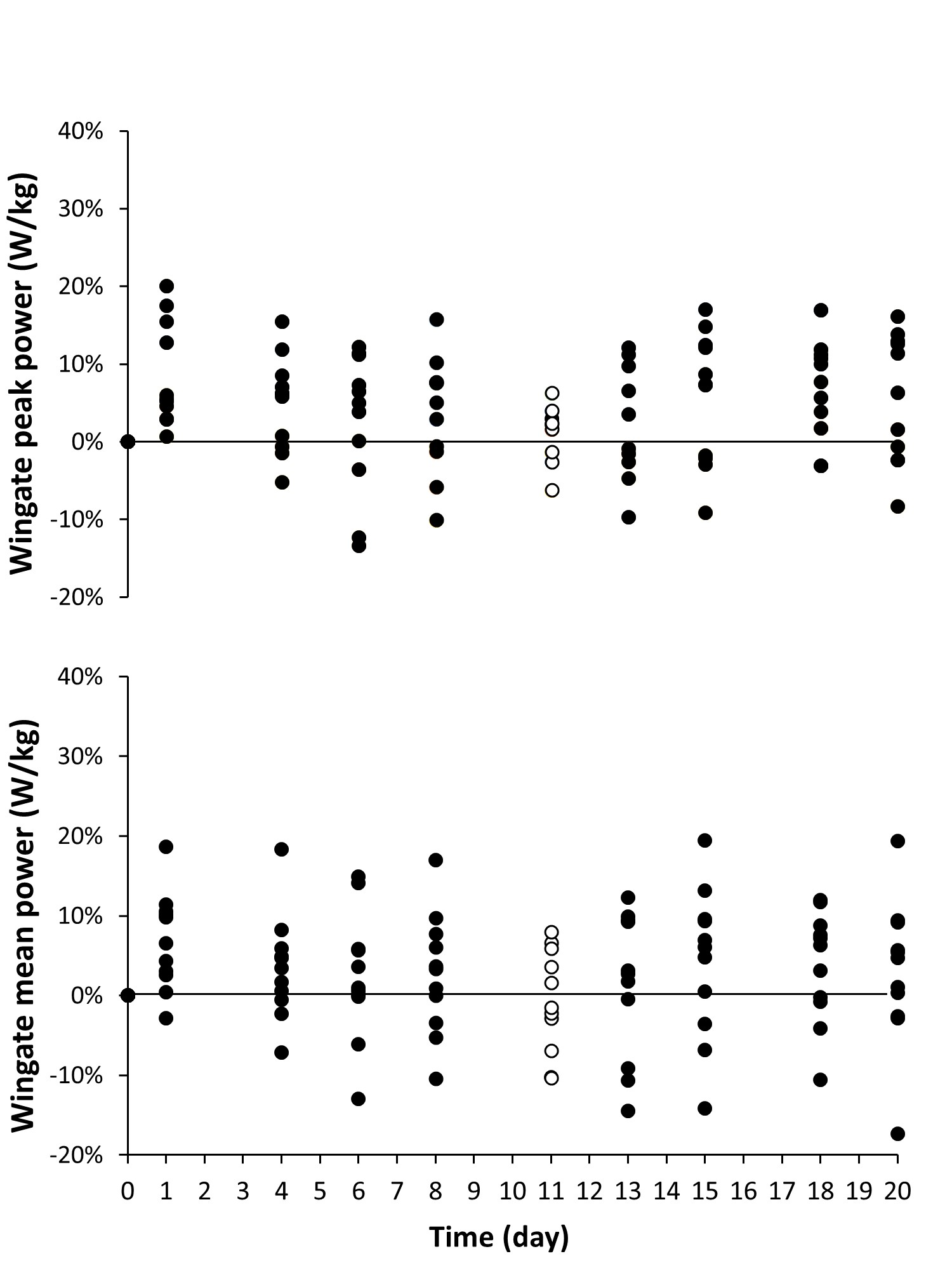

Supplement: S2 Fig — Each dot represents the caffeine vs placebo change in each individual and positive values indicate a superior performance with caffeine over placebo. (TIF) [file pone.0210275.s002.tif]
